# Recent advances in cell sheet technology for bone and cartilage regeneration: from preparation to application

**DOI:** 10.1038/s41368-019-0050-5

**Published:** 2019-05-21

**Authors:** Yuezhi Lu, Wenjie Zhang, Jie Wang, Guangzheng Yang, Shi Yin, Tingting Tang, Chunhua Yu, Xinquan Jiang

**Affiliations:** 1 Department of Prosthodontics, Shanghai Ninth People’s Hospital, College of Stomatology, Shanghai Jiao Tong University School of Medicine; National Clinical Research Center for Oral Diseases; Shanghai Engineering Research Center of Advanced Dental Technology and Materials; Shanghai Key Laboratory of Stomatology & Shanghai Research Institute of Stomatology, Shanghai, China; 2 Shanghai Key Laboratory of Orthopaedic Implants, Department of Orthopaedic Surgery, Shanghai Ninth People’s Hospital, Shanghai Jiao Tong University School of Medicine, Shanghai, China

**Keywords:** Oral diseases, Cell biology, Rehabilitation

## Abstract

Bone defects caused by trauma, tumour resection, infection and congenital deformities, together with articular cartilage defects and cartilage–subchondral bone complex defects caused by trauma and degenerative diseases, remain great challenges for clinicians. Novel strategies utilising cell sheet technology to enhance bone and cartilage regeneration are being developed. The cell sheet technology has shown great clinical potential in regenerative medicine due to its effective preservation of cell–cell connections and extracellular matrix and its scaffold-free nature. This review will first introduce several widely used cell sheet preparation systems, including traditional approaches and recent improvements, as well as their advantages and shortcomings. Recent advances in utilising cell sheet technology to regenerate bone or cartilage defects and bone–cartilage complex defects will be reviewed. The key challenges and future research directions for the application of cell sheet technology in bone and cartilage regeneration will also be discussed.

## Introduction

Bone defects caused by various aetiologies, such as trauma, tumours, infection and congenital deformities, together with articular cartilage defects and osteochondral complex defects caused by trauma and degenerative diseases, are common clinical diseases that significantly affect the patient’s quality of life. Repair and regenerating these defects in bone and cartilage is a considerable challenge for clinicians.^1–4^ There has been significant progress in the development of tissue engineering over the past two decades, which has brought new hope for the regenerative treatment of bone and cartilage defects.^5,6^ Conventional tissue engineering techniques mainly include the injection of a cell suspension and the transplantation of scaffolds seeded with cells.^7^ However, several problems remain to be solved. With the injection of a cell suspension, locating the injected suspension and controlling the shape and size of the cell suspension after injection is difficult. The number of cells that can be delivered by one injection is quite limited, and the cells are easily lost after injection. Additionally, a uniform distribution of the injected suspension is difficult to achieve. Thus far, the cell injection technique cannot meet the requirements for regenerating tissue morphology and function. An ideal biodegradable scaffold material that can efficiently promote cell adhesion, proliferation and extracellular matrix (ECM) secretion with suitable mechanical properties is still being sought by researchers.^8^ Existing scaffold materials usually have several limitations, such as insufficient biological activity, unstable degradation rate and immunogenicity, resulting in immune responses and inflammation after transplantation. Cell–material interactions are usually uncontrollable and may result in high cell mortality.^9^ Cell–cell interactions and ECM formation contribute to maintaining tissue stability. Conventional tissue engineering techniques for harvesting cells by trypsin digestion damage cell–cell interactions, cell–ECM interactions and cell membrane proteins, resulting in decreased cell adhesion and proliferation.

To overcome the shortcomings of conventional tissue engineering technology, cell sheet technology, an alternative approach, has gradually attracted the attention of researchers in recent years. Cell sheet technology was developed based on a novel technique for culturing and harvesting cells using temperature-responsive culture dishes, which was first reported in 1990.^10,11^ The hydrophilic and hydrophobic properties of the temperature-sensitive material poly(*N*-isopropylacrylamide) (PIPAAm) could be altered by changing the temperature, resulting in control over cell attachment and detachment.^12^ Cell sheet technology can be used to harvest cells without utilising proteolytic enzymes, such as trypsin, or chelating agents, such as ethylenediaminetetraacetic acid. Thus the cell–cell junctions, ECM and cell sheet structure are effectively preserved, allowing the constructed tissue to have a high cell density and a uniform cell distribution and thus to mimic native tissue more closely. In addition, cell sheets are prepared by the formation of cell–cell junctions and the secretion of ECM and are free from the limitations of scaffold materials, such as the immune and inflammatory reactions caused by scaffold implantation, tissue collapse caused by a fast degradation rate and compromised tissue formation caused by a slow degradation rate.^13–18^ The application of this technology in bone and cartilage regeneration has been widely studied. On the one hand, cell sheets can be used without scaffolds for bone and cartilage regeneration; thus they more closely mimic native tissue and avoid the limitations and potential problems of scaffolds.^19,20^ On the other hand, cell sheets can also be used in combination with various scaffolds and may be a better choice than traditional scaffolds seeded with cell suspensions because cell sheets can effectively preserve cell–cell junctions and ECM.^21,22^

Several widely used cell sheet preparation systems, including traditional methods, and recent improvements in these methods, as well as their advantages and shortcomings, will be reviewed. Recent advances in the application of cell sheet technology for the repair and regeneration of bone and cartilage defects will also be reviewed. Furthermore, the key limitations of cell sheet applications in bone and cartilage regeneration, along with directions for future research, will be discussed.

## Preparation of cell sheets

A variety of systems can be used to construct cell sheets, including temperature-responsive, electro-responsive, photo-responsive, pH-responsive, mechanical, and magnetic systems.^18,23^ With continuous advances in cell sheet technology in recent years, approaches to optimising the preparation of cell sheets have been proposed based on these systems (Table 1).

**Table 1:** Summary of cell sheet preparation systems

Author	Preparation system	Critical approach	Preparation of cell sheets			Ref.
			Cell sheet formation	Cell sheet detachment	Shape and structure	
Hatakeyama	Temperature-responsive	INS immobilisation	Proliferation	Within 2 h at 20 ℃	Monolayer	^27^
Ebara	Temperature-responsive	RGDS immobilisation	Adhesion	Within 90 min at 20 ℃	Monolayer	^28^
Kwon	Temperature-responsive	PIPAAm-PM		30 min at 20 ℃	Monolayer	^29^
Kwon	Temperature-responsive	PIPAAm(PEG)-PM		19 min at 20 ℃	Monolayer	^30^
Ebara	Temperature-responsive	P(IPAAm-co-CIPAAm)		35 min at 20 ℃	Monolayer	^31^
Guo	Temperature-responsive	Temperature and saccharides dual-responsive		Within 30 min at 20 ℃	Monolayer	^32^
Patel	Temperature-responsive	PIPAAm-APTES	Adhesion and proliferation	Within 2.5 min	Monolayer	^33^
Hatakeyama	Temperature-responsive	EB-induced pattern and RGDS–INS immobilisation	Adhesion and proliferation	More than 3 h at 20 ℃	Patterned monolayer	^36^
Tsuda	Temperature-responsive	EB-induced patterned P(IPAAm–BMA)			Patterned coculture monolayer	^37^
Isenberg	Temperature-responsive	PDMS mold			Micropatterned monolayer	^38^
Isenberg	Temperature-responsive	PDMS mold			Micropatterned monolayer	^39^
Lin	Temperature-responsive	PDMS mold			Micropatterned monolayer	^40^
Williams	Temperature-responsive	Microcontact printed with FN		Within 2 h at 20 ℃	Micropatterned aligned monolayer	^41^
Hannachi	Temperature-responsive	Microcontact printed with FN			Micropatterned cocultured monolayer	^42^
Guillaume-Gentil	Electro-responsive	Polyelectrolyte			Monolayer	^45^
Inaba	Electro-responsive	Alkanethiol SAM-RGD		Within 10 min	Monolayer	^46^
Seto	Electro-responsive	Oligopeptide		Above 90% within 7 min	Monolayer	^47^
Mochizuki	Electro-responsive	Oligopeptide		Within 10 min	Monolayer	^48^
Kakegawa	Electro-responsive	Oligopeptide		5 min	Patterned monolayer	^49^
Guillaume-Gentil	Electro-responsive	Photolithography and polyelectrolyte			Micropatterned cocultured monolayer	^50^
Enomoto	Electro-responsive	Oligopeptide and porous membrane substrate	Oxygen and nutrients	7 min	Monolayer and multilayer (stacked)	^51^
Hong	Photo-responsive	Ultraviolet and TiO_2_ nanodot-coated quartz		Above 90% at 20 min	Monolayer	^53^
Zhu	Photo-responsive	Ultraviolet and PDA/TiO_2_ film		Above 90% at 20 min	Monolayer	^54^
Yu; Cheng	Photo-responsive	Ultraviolet and TiO_2_ nanodot film-RGD	Adhesion	30 min	Monolayer	^55, 56^
Jiang	Photo-responsive	Ultraviolet and laminin-521	Adhesion and proliferation	30 min	Monolayer	^57^
Liu	Photo-responsive	Ultraviolet and TiO_2_ nanodot film and GelMA			Micropatterned multilayer	^58^
Wang	Photo-responsive	Visible light and Si(p/n)		10 min	Monolayer	^59^
Na	Photo-responsive	Near infrared and PEDOT and micropatterned optical lens		within 5 min	Patterned monolayer	^60^
Guillaume-Gentil	pH-responsive	PAH-PSS-ITO		10–20 min	Monolayer	^61^
Kim	Mechanical	Gelatin	Proliferation and ECM		Monolayer (3–4 cell layers)	^63^
Ito	Magnetic	MCLs		Remove the magnet	Multilayer	^65^
Ito	Magnetic	RGD-MCLs	Adhesion	Remove the magnet		^66^
Ito	Magnetic	MCLs		Remove the magnet	Cocultured multilayer	^67^
Zhang	Magnetic	nGO@Fe_3_O_4_ MNPs		Remove the magnet	Precisely controlled multilayer	^68^

APTES 3-aminopropyltriethoxysilane; BMA n-butyl methacrylate; CIPAAm 2-carboxyisopropylacrylamide; EB electron beam; ECM extracellular matrix; FN fibronectin; GelMA gelatine methacrylate; INS immobilising insulin; IPAAm N-isopropylacrylamide; ITO indium tin oxide; MCL magnetite cationic liposome; MNP magnetic nanoparticle; PAH poly(allylamine hydrochloride); PDA polydopamine; PDMS polydimethylsiloxane; PEDOT poly(3,4-ethylenedioxythiophene); PEG poly(ethylene glycol); PM porous membrane; PIPAAm poly(*N*-isopropylacrylamide); PSS, poly(styrene sulfonate); RGD Arg-Gly-Asp; RGDS Arg-Gly-Asp-Ser; SAM self-assembled monolayer

### Temperature-responsive systems

The first proposed systems were temperature-responsive systems, which are currently the most widely used for cell sheet preparation.^24^ The temperature-sensitive material PIPAAm, which has a critical temperature of 32 °C, is covalently bound to the bottom of a cell culture dish. When cells are cultured in the typical 37 °C environment, PIPAAm exhibits hydrophobic properties, which facilitate cell adhesion and proliferation to promote cell sheet formation. When the temperature is below 32 °C, PIPAAm exhibits hydrophilic properties, and the adhered cell sheet can be completely separated from the culture dish via the formation of a hydration layer between the surface of the culture dish and the cell sheet.^25^ Many improvements to traditional temperature-responsive systems have been proposed recently.^26^

To accelerate the formation of cell sheets, biomolecules can be utilised to promote cell adhesion or proliferation. Immobilising insulin (INS) on temperature-responsive culture dishes shortened the period of cell sheet formation by promoting cell proliferation,^27^ and immobilising the synthetic cell adhesion peptide Arg-Gly-Asp-Ser (RGDS) on temperature-responsive culture dishes accelerated cell sheet formation by promoting cell adhesion.^28^

Several improved systems have been developed to shorten the cell sheet detachment time, which could permit further complex manipulation of cell sheets and prevent potential damage to cell viability from long exposures to low, non-physiological temperatures. PIPAAm was grafted onto a porous membrane (PM) by electron beam (EB) irradiation to form a PIPAAm-PM substrate for cell sheet preparation. Compared with the traditional PIPAAm substrate, the PIPAAm-PM substrate showed a decrease in cell sheet detachment time from approximately 75 to 30 min at 20 °C.^29^ Furthermore, the co-grafting of poly(ethylene glycol) (PEG) with PIPAAm onto the PM by EB irradiation to form a PIPAAm(PEG)-PM substrate was proposed and ultimately shortened the cell sheet detachment time to 19 min at 20 °C.^30^ 2-Carboxyisopropylacrylamide (CIPAAm) was synthesised with a similar side chain structure as that of *N*-isopropylacrylamide (IPAAm) and a functional carboxylate group. A P(IPAAm-co-CIPAAm) substrate was developed to accelerate cell sheet detachment by reducing the exposure time to 35 min at 20 °C.^31^ A temperature and saccharide dual-responsive system consisting of both PIPAAm and the saccharide-sensitive material poly(3-acrylamidophenylboronic acid) was proposed for cell sheet preparation. Cell sheets could be harvested more efficiently by simultaneously reducing the temperature and increasing the saccharide concentration. The exposure time at 20 °C for cell sheet detachment was shortened to within 30 min in 10 g/L fructose.^32^ A small amount of 3-aminopropyltriethoxysilane (APTES) was utilised to develop a PIPAAm-APTES substrate, which remarkably shortened the detachment time of cell sheets to within 2.5 min in cold culture medium and improved cell adhesion and proliferation simultaneously.^33^

The incorporation of specific structures into cell sheets is of great importance for mimicking the complex architecture of native tissues and has drawn the attention of researchers. Cell patterning or micropatterning technology has been applied in cell sheet preparation systems.^34,35^ A patterned, bio-functional and temperature-responsive system was fabricated using EB patterning and appropriate metal masks, resulting in the formation of a surface pattern of carboxyl-functional thermo-responsive polymers; this process was followed by site-selective bio-functionalisation with RGDS-INS biomolecules. Cell sheet formation using this system was improved by the promotion of cell adhesion and proliferation, but the detachment time was prolonged to >3 h at 20 °C.^36^ A dual temperature-responsive substrate for the preparation of patterned cell sheets containing two different cell types was developed via the patterned polymerisation of n-butyl methacrylate into PIPAAm using EB irradiation.^37^ A temperature-responsive system for the preparation of micropatterned cell sheets was proposed by Isenberg et al. The polystyrene substrate for cell sheet preparation was micropatterned by hot embossing with polydimethylsiloxane moulds containing an array of parallel grooves and then grafted with PIPAAm.^38^ A similar micropatterned substrate was used to prepare monolayered cell sheets with micropatterns.^39,40^ Williams et al. developed a temperature-responsive system combined with a microcontact printing technique for the preparation of aligned cell sheets. Lanes were formed on the PIPAAm substrate by microcontact printing of fibronectin (FN) using a patterned stamp. Cells incubated in a serum-free environment preferentially adhered to the lanes containing FN, resulting in ordered alignment. With the addition of serum, the cells expanded into the regions without FN to form a cell sheet. A patterned monolayered cell sheet was then harvested by reducing the temperature.^41^ A micropatterned and co-cultured cell sheet preparation system was developed by microcontact printing of FN, followed by the seeding of one type of cell in serum-free conditions and the seeding of another type of cell in the presence of serum.^42^

For application in bone and cartilage regeneration, commercial temperature-responsive culture dishes were used to culture cells, such as mesenchymal stem cells (MSCs), chondrocytes and synovial cells. After the cells were cultured for several days or weeks to form cell sheets, the temperature-responsive culture dishes were exposed to 20 °C or room temperature for a period of time, and polyvinylidene fluoride (PVDF) membranes or forceps were used to harvest the cell sheets.^20,21,43,44^

### Electro-responsive systems

A new electro-responsive system for cell sheet preparation was proposed by Guillaume-Gentil et al. The cell sheet was formed by growing cells to confluence on a thin polyelectrolyte film. Then the cell sheet was detached from the polyelectrolyte substrate with electrochemical control, such as by applying a positive potential for electrochemical polarisation, or spontaneously without electrochemical control.^45^ Inaba et al. proposed another electro-responsive system for cell sheet preparation involving a gold surface modified with a self-assembled monolayer of alkanethiol and Arg-Gly-Asp (RGD) peptides. Cell sheets formed on the substrate and then could be harvested within 10 min by the application of a negative electrical potential.^46^ To alleviate the problem of potentially harmful chemicals used in the electro-responsive system remaining in the cell sheets, leading to an inflammatory response upon application, an oligopeptide containing a cell-adhesion domain, RGD, in the centre and cysteine residues at both terminals was utilised to modify the gold surface. The cell sheets were also detached by the application of a negative electrical potential.^47^ Similar electro-responsive systems for cell sheet preparation were developed using oligopeptides and a negative electrical potential.^48,49^ Cell micropatterns could be transferred to a hydrogel by means of cell-adhesive and cell-repulsive micropatterned oligopeptides.^49^ An electrochemically switchable system for the preparation of micropatterned heterotypic cell sheets was proposed; the system combined photolithographic processing and the local electrochemical dissolution of polyelectrolytes.^50^ A porous gold-coated membrane substrate that improved oxygen and nutrient supply was covered with an oligopeptide layer and utilised as an electro-responsive system for cell sheet preparation. A monolayered cell sheet could be detached in 7 min by the application of a negative electrical potential, and multilayered structures could be formed by subsequent stacking.^51^ Most studies have mainly focused on electro-responsive systems for cell sheet preparation for application in vascularised tissues, and the application of such systems in bone and cartilage regeneration has not been reported thus far.^47,48,52^ The specific culture substrates and devices needed in the electro-responsive system might be obstacles for widespread application.

### Photo-responsive systems

**Fig. 1:**
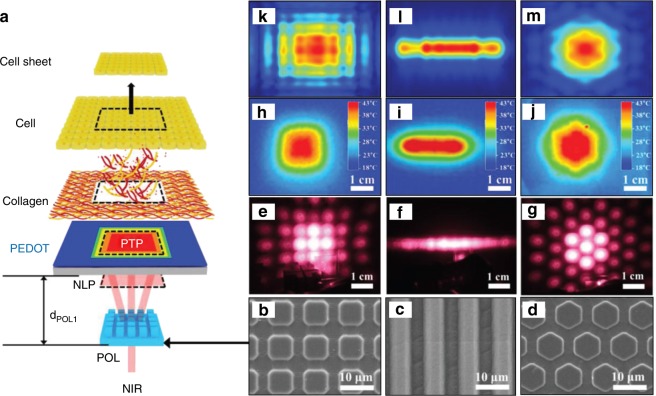
A photo-responsive system for the preparation of micropatterned cell sheets. **a** A schematic illustration for the formation of an near infrared (NIR) light pattern (NLP), a photothermal pattern (PTP), and harvesting of a square-type cell sheet using an optical setup including an NIR laser and patterned optical lens (POL). **b**–**d** Field-emission scanning electron microscopic images for micropatterned POLs with **b** square, **c** linear, and **d** hexagonal patterns. **e**–**g** Photographic images of NLPs observed by a digital camera equipped with a visible filter generated from the **e** square, **f** line, and **g** hexagon POLs of **b**–**d**, respectively. **h**–**j** Thermal images obtained for the PTP on the PEDOT substrate generated from the corresponding NLP of **e**–**g**, respectively. **k**–**m** The finite-difference time-domain calculation result of NLPs using the experimental parameter of the corresponding POL of **b**–**d**, respectively (adapted from ref. ^60^ with permission)

A photo-responsive system for cell sheet preparation based on ultraviolet light-mediated changes in the hydrophilicity and hydrophobicity of the culture substrate was proposed by Hong et al. The cells were cultured on a titanium dioxide (TiO_2_) nanodot-coated quartz substrate and formed a cell sheet. More than 90% of the cells could be detached from the substrate by the application of 365-nm ultraviolet illumination for 20 min.^53^ A polystyrene substrate covered with a composite polydopamine/TiO_2_ film was applied for cell sheet preparation. The application of 365-nm ultraviolet illumination for 20 min allowed the detachment of >90% of fibroblasts and >77% of osteoblasts from the substrate.^54^ A TiO_2_ nanodot film with surface-immobilised RGD was proposed for cell sheet preparation and was shown to promote cell sheet formation by improving cell adhesion, and 365-nm ultraviolet illumination for 30 min prompted cell sheet detachment.^55,56^ Immobilising human recombinant laminin-521 on a TiO_2_ nanodot film could promote cell sheet formation by improving cell adhesion and proliferation, and these cell sheets could be harvested by 365-nm ultraviolet illumination for 30 min.^57^ A photo-responsive system for the preparation of micropatterned multilayered cell sheets was developed using a TiO_2_ nanodot film and photo-cross-linkable gelatine methacrylate (GelMA). Photomask-assisted 254-nm ultraviolet illumination was used to micropattern a TiO_2_ nanodot film, and 365-nm ultraviolet illumination was used to harvest the cell sheet. The photo-cross-linkable GelMA was used to transfer and stack monolayer cell sheets into a multilayer structure.^58^ A visible light-responsive system for cell sheet preparation was proposed by Wang et al. The cell sheets could be detached from silicon wafer substrates with p/n junctions (Si(p/n)) after 10 min of illumination with low-energy visible light.^59^ Na et al. proposed a photo-responsive system for cell sheet preparation based on near infrared (NIR) light, which is highly transmissive through tissues and safer at an appropriate intensity than ultraviolet and visible light. Owing to the high photothermal efficiency of the poly(3,4-ethylenedioxythiophene) (PEDOT) substrate and the photothermal pattern formed by the diffraction of NIR light through a micropatterned optical lens, cell sheets with various patterns could be harvested within 5 min (Fig. 1).^60^

### pH-responsive systems

A pH-responsive system for cell sheet preparation was proposed by Guillaume-Gentil et al. Human placenta-derived MSCs were seeded on a pH-responsive substrate, which was obtained via layer-by-layer deposition of cationic poly(allylamine hydrochloride) and anionic poly(styrene sulfonate) on conductive indium tin oxide electrodes. Then a monolayered cell sheet could be harvested by decreasing the local or global pH.^61^ However, the change in pH might be detrimental to the physical conditions of the cells; thus this type of system is rarely applied in cell sheet preparation.^18^

### Mechanical systems

Mechanical systems may be easy to prepare a cell sheet without specific culture substrates or techniques, but the required manipulation can be challenging. While few studies have been conducted on simple mechanical techniques, these techniques have been widely used to prepare cell sheets for application. To form an osteogenic or chondrocytic cell sheet, MSCs or chondrocytes were cultured in osteogenic or chondrogenic medium for several days or weeks.^62–64^ When the cells formed a contiguous cell sheet, a cell scraper could be used to lift the cell sheet inward with gentle scraping from the periphery in phosphate-buffered saline.^19^ Forceps have also been used to mechanically peel off and detach cell sheets.^62^ A novel mechanical system for the preparation of osteogenic cell sheets involving the addition of gelatine to osteogenic medium was proposed. Compared to the conventional osteogenic cell sheet, the gelatine-induced osteogenic cell sheet had great cell proliferation and abundant ECM, resulting in a thick and strong cell sheet.^63^

### Magnetic systems

A magnetic system for cell sheet preparation was first introduced by Ito et al. Magnetite cationic liposomes (MCLs) with a positive surface charge for improved adsorption were taken up by cells. Multilayered sheets of MCL-labelled cells were formed in an ultralow-attachment plate via magnetic attraction. The cell sheets were detached from the plates by removing the magnetic field and then harvested with a magnet.^65^ A culture surface coated with RGD peptide-conjugated MCLs (RGD-MCLs) by magnetic attraction was proposed for cell sheet preparation. A contiguous cell sheet containing RGD-MCLs was formed by incubating cells on the RGD-MCL substrate, which facilitated cell adhesion, and was then detached by removing the magnet.^66^ Multilayered cell sheets containing heterotypic co-cultured cells labelled with MCLs were fabricated by the application of magnetic force.^67^

**Fig. 2:**
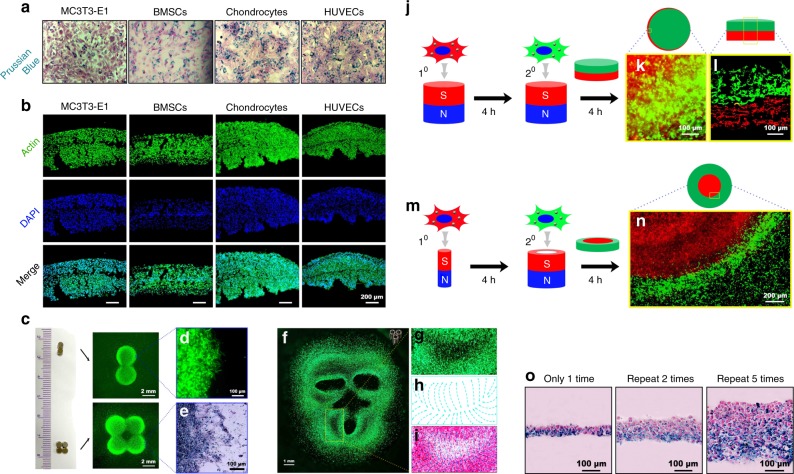
A magnetic system for cell sheet preparation based on the cellular uptake of nGO@Fe_3_O_4_. **a** MC3T3-E1 cells, bone marrow-derived mesenchymal stem cells (BMSCs), chondrocytes, and human umbilical vein endothelial cells (HUVECs) were incubated with nGO@Fe_3_O_4_ magnetic nanoparticles (MNPs). Prussian blue staining was used to visualise the distribution of the MNPs. The **b** MC3T3-E1 cells, BMSCs, chondrocytes, and HUVECs were attracted by the magnetic force to form multilayer cell sheets. **c** The magnet pattern controlled the shape of the cell sheets. **d** Cell accumulation was observed at the marginal region of the cell sheet. **e** The marginal region of the cell sheet was observed after 24 h of culture with Prussian blue staining. **f** A monkey face-like cell sheet pattern was fabricated via three hollow cylinder magnets. The local region surrounded by the yellow rectangular frame is magnified and presented in **g**. **h** The cells were arranged in regular continuous curves. **i** A merged image of the cell distribution and the characteristic curves. **j** Schematic illustration of the fabrication strategy to form bilayer cell sheets. **k** The accumulation of GFP+ cells on the RFP+ cell sheet was observed. **l** The bilayer cell sheets were observed after 24 h of culture. **m** Schematic illustration of the strategy to fabricate inlaid cell sheets. **n** A clear boundary line between the GFP+ cells and the RFP+ cells was observed. **o** Prussian-blue-stained paraffin sections of cell sheets fabricated via repeated cell seeding. (Adapted from ref. ^68^ with permission.)

Recently, our group developed a magnetic system for preparing cell sheets based on the cellular uptake of Fe_3_O_4_ magnetic nanoparticles (MNPs) coated with nanoscale graphene oxide (nGO@Fe_3_O_4_). The nGO@Fe_3_O_4_ MNPs could be easily and rapidly taken up by dental pulp stem cells (DPSCs), MC3T3-E1 cells, bone marrow-derived mesenchymal stem cells (BMSCs), chondrocytes and human umbilical vein endothelial cells (HUVECs) (Fig. 2a). Multilayered cell sheets were easily and rapidly constructed via magnetic attraction (Fig. 2b). In addition to the advantage of easy and rapid preparation of multilayered cell sheets, the magnetic system could precisely control cell sheet shape and structure. Cell sheets with different shapes were formed using two or four magnets (Fig. 2c). A monkey face-like cell sheet pattern was formed using three hollow cylindrical magnets (Fig. 2d). The separate addition of two types of cells resulted in a cell sheet with a bilayered structure (Fig. 2e). In addition, one cell sheet could be inlaid within another cell sheet by simultaneously changing both the cell type and magnet pattern (Fig. 2f). Moreover, the cell sheet thickness could be controlled by the repeated addition of cells (Fig. 2g).^68^

## Application of cell sheet technology in bone and cartilage regeneration

With the rapid development of cell sheet technology, cell sheets have been applied in the regeneration of multiple tissues and organs, such as the heart, liver, kidney, cornea, bladder, oesophagus, trachea, tendon, periodontium, bone and cartilage.^69–76^ In this review, we focus on recent progress in the application of cell sheet technology in bone and cartilage regeneration (Table 2). Although various systems have been developed for cell sheet preparation, temperature-responsive and mechanical systems are the most widely used systems to prepare cell sheets for bone and cartilage regeneration. For the application of temperature-responsive systems in preparing cell sheets, commercial temperature-responsive culture dishes were used to culture cells, such as MSCs, chondrocytes and synovial cells. Cell sheets formed after several days or weeks in culture in temperature-responsive culture dishes and were harvested using PVDF membranes or forceps upon exposure to 20 °C or room temperature for a period time.^20,21,43,44^ With regard to the preparation of cell sheets using mechanical systems, MSCs, HUVECs or chondrocytes were cultured in induction medium for several days or weeks to form contiguous cell sheets; then cell scrapers or forceps were utilised to lift or peel off the cell sheets.^62,64^

**Table 2:** Summary of the application of cell sheet technology in the bone and cartilage regeneration in vivo

Author	Cell type	Preparation system	Scaffold/growth factor	In vivo	Function	Ref.
Akahane	Rat BMSCs (osteogenic induction)	Mechanical	None	Rats (subcutaneous)	Osteogenesis	^19^
Ma	Rabbit BMSCs (osteogenic induction)	Mechanical	None	Nude mice (subcutaneous)	Osteogenesis	^77^
Shimizu	Human MSCs	Magnetic	None	Nude rats (cranial defects)	Osteogenesis	^78^
Ueyama	Rat BMSCs (osteogenic induction)	Mechanical	None	Rats (mandibular symphysis)	Osteogenesis	^79^
Nakamura	Rat BMSCs (osteogenic induction)	Mechanical	None	Rats (femoral fracture)	Osteogenesis	^81^
Shimizu	Rat BMSCs (osteogenic induction)	Mechanical	None	Rats (femoral fracture)	Osteogenesis	^82^
Zhou	Porcine BMSCs (osteogenic induction)	Mechanical	PCL-CaP	Nude rats (subcutaneous)	Osteogenesis	^84^
Ma	Rabbit BMSCs (osteogenic induction)	Mechanical	β-TCP	Rabbits (subcutaneous and mandibular fracture)	Osteogenesis	^83^
Ueha	Rat BMSCs (osteogenic induction)	Mechanical	β-TCP	Rats (femoral defects)	Osteogenesis	^85^
Lin	Rat BMSCs (osteogenic induction)	Mechanical	β-TCP/COL-I	Nude mice (subcutaneous)	Osteogenesis	^86^
Akahane	Old rat BMSCs (osteogenic induction)	Mechanical	β-TCP	Rats (subcutaneous)	Osteogenesis	^87^
Xie	Human ESMSCs (osteogenic induction)	Temperature-responsive	PSeD	Rats (calvarial defects)	Osteogenesis	^21^
Shan	Canine BMSCs (osteogenic induction)	Temperature-responsive	PLGA	Canine (mandibular defects)	Osteogenesis	^88^
Liu	Rat BMSCs (osteogenic induction)	Not mentioned	CBB	Osteoporotic rats (calvarial defects)	Osteogenesis	^89^
Long	Mouse BMSCs	Temperature-responsive	Devitalised allograft	Mice (femoral defects)	Osteogenesis	^90^
Qi	Rat BMSCs	Mechanical	CaP/PRP	Rats (femoral defects)	Osteogenesis	^92^
Dang	Human BMSCs	Not mentioned	TGF-β1 and BMP-2	Rats (calvarial defects)	Osteogenesis	^94^
Ren	Human BMSCs and HUVECs	Mechanical	None	Nude mice (subcutaneous)	Osteogenesis and angiogenesis	^96^
Ren	Human BMSCs (undifferentiated and osteogenic) and HUVECs	Mechanical	None	Nude mice (subcutaneous)	Osteogenesis and angiogenesis	^97^
Mendes	Human BMSCs (osteogenic and CD146 pericytes) and HUVECs	Temperature-responsive	TGF-β1	Nude mice (subcutaneous)	Osteogenesis and angiogenesis	^99^
Zhang	Rabbit adipose-derived MSCs (osteogenic and endothelial induction)	Mechanical	CHA	Nude mice (subcutaneous)	Osteogenesis and angiogenesis	##
Nakano	Rat BMSCs (osteogenic induction)	Mechanical	β-TCP	Rats (subcutaneous)	Osteogenesis and angiogenesis	##
Ma	Rabbit BMSCs (osteogenic induction)	Mechanical	β-TCP	Rabbits (muscular pockets)	Osteogenesis and angiogenesis	##
Kang	Human MSCs (undifferentiated and osteogenic) and HUVECs	Mechanical	β-TCP	Nude mice (subcutaneous)	Osteogenesis and angiogenesis	^62^
Zhang	Rat BMSCs (undifferentiated and endothelial and osteogenic induction)	Mechanical	β-TCP	Rats (calvarial defects)	Osteogenesis and angiogenesis	##
Kaneshiro	Rabbit chondrocytes (multilayer)	Temperature-responsive	None	Rabbits (femoral condyle)	Chondrogenesis	##
Yano	mouse/canine chondrocytes and human BMSCs	Temperature-responsive	TD-198946	Mice/canine/NODSCID mice (femoral condyle)	Chondrogenesis	##
Ebihara	Minipig chondrocytes (multilayer)	Temperature-responsive	None	Minipigs (femoral condyle)	Chondrogenesis	^43^
Ito	Rabbit chondrocytes and synovial cells (multilayer)	Temperature-responsive	None	Rabbits (femoral patellar groove)	Chondrogenesis and osteogenesis	^44^
Wang	Minipig chondrocytes and BMSCs	Mechanical	PCL/HA	Nude mice (subcutaneous)	Chondrogenesis and osteogenesis	^64^
Wang	Minipig chondrocytes and BMSCs	Mechanical	PCL/HA	Minipigs (subcutaneous and intramuscular)	Chondrogenesis and osteogenesis	^22^
Zhang	Human DPSCs	Magnetic	BMP-2 and TGF-β3	Nude mice (subcutaneous)	Osteogenesis and angiogenesis and chondrogenesis	^68^

BMP-2 bone morphogenetic protein 2; BMSC bone marrow-derived mesenchymal stem cell; CaP calcium phosphate; DPSC dental pulp stem cell; HA hydroxyapatite; HUVEC human umbilical vein endothelial cell; MSC mesenchymal stem cell; PCL polycaprolactone; PRP platelet-rich plasma; TCP tricalcium phosphate; TGF transforming growth factor

### Progress in the application of cell sheet technology in bone regeneration

The application of cell sheets alone in ectopic sites or bone defects has shown promising results. After osteogenic induction, rat BMSCs were prepared into cell sheets using a mechanical system and then subcutaneously transplanted into the rat thigh. New bone formation was promoted without scaffolds.^19^ In another study, the mechanical system was used to prepare cell sheets of rabbit BMSCs, and then these sheets were transplanted into subcutaneous pockets of nude mice. The formation of new bone without the use of scaffold was facilitated by BMSC sheets alone.^77^ The transplantation of multilayered human MSC sheets prepared by the magnetic system into cranial bone defects in nude rats resulted in the formation of new bone surrounded by osteoblast-like cells in the defects.^78^ The preparation of osteogenic cell sheets by a mechanical system and the transplantation into the site of maxillofacial bone defects using a rat mandibular symphysis model enhanced new bone formation.^79^ Fracture healing is normally a spontaneous process that involves initial inflammation, followed by callus formation and finally bone remodelling. However, in some cases, delayed bone union and nonunion may occur due to complex factors, such as disruptions in the biological or mechanical environment.^80^ The application of cell sheets alone in these situations of compromised fracture healing has also shown promising results. A series of studies examined the use of osteogenic cell sheets of rat BMSCs created using mechanical systems for the treatment of fracture nonunion and critical fracture healing. The transplantation of osteogenic cell sheets into fractured rat femurs enhanced bone formation at the fracture site and resulted in stable bone union.^81^ The injection of osteogenic cell sheets into a rat femoral critical fracture healing model enhanced bone regeneration and finally led to bone union.^82^ Ma et al. wrapped osteogenic pre-differentiated rabbit BMSC sheets around a 2-mm mandibular fracture gap and observed an improvement in delayed bone fracture healing and a reduction in the amount of fibrous tissue at the fracture site.^83^

Intact cell sheet formation relies on cell–cell connections and the ECM secreted by cells. However, as cell sheets have poor mechanical properties, repairing bone defects with cell sheets alone remains a challenge. Thus, many studies have investigated the repair of bone defects by combining cell sheets prepared by mechanical or temperature-responsive systems with scaffolds, which could provide initial mechanical and spatial support. In a study by Zhou et al., multilayered porcine BMSC sheets were harvested after 1–2 weeks of osteogenic induction and then wrapped around pre-seeded polycaprolactone–calcium phosphate (PCL-CaP) scaffolds to form cell sheet scaffold constructs.^84^ New cortical and cancellous bone formed within the cell sheet scaffold constructs after subcutaneous implantation into nude rats. Ma et al. wrapped osteogenic pre-differentiated cell sheets derived from rabbit BMSCs around porous beta-tricalcium phosphate (β-TCP) scaffolds. Subcutaneous implantation of these cell sheet scaffold constructs into rabbits resulted in superior ectopic bone formation.^83^ β-TCP scaffolds containing BMSCs were wrapped in osteogenic matrix BMSC sheets to form BMSC/TCP/sheet constructs. The subcutaneous implantation of these constructs, as well as their implantation into femoral bone defects, showed very promising osteogenic potential.^85^ Porous β-TCP/COL-I composite scaffolds wrapped in BMSC sheets after osteogenic induction implanted in nude mice have also shown superior osteogenic ability.^86^ BMSC sheets from both young and old rats after osteogenic induction were combined with β-TCP scaffolds and transplanted subcutaneously into recipient rats; bone formation was promoted in both the young and old BMSC sheets groups, indicating that BMSC sheets from old donors were also capable of bone regeneration.^87^ In addition to the most widely used β-TCP ceramic scaffold, polymeric scaffolds, including poly(sebacoyl diglyceride) (PSeD) and poly(lactic-co-glycolic acid) (PLGA), combined with cell sheets have also been studied for bone regeneration. Xie et al. wrapped human ethmoid sinus mucosal membrane-derived MSC sheets around porous PSeD scaffolds pre-seeded with rat BMSCs and showed that implantation of these constructs into 8-mm-diameter critical-sized calvarial defects in rats promoted new bone regeneration.^21^ The utilisation of PLGA scaffolds wrapped in BMSC sheets after osteogenic induction also improved bone regeneration in canine mandibular bone defects.^88^ Furthermore, scaffolds derived from natural bone, including calcined bovine bones and allografts, have been studied. Liu et al. wrapped rat BMSCs after osteogenic induction around calcined bovine bones (CBBs) to form CBB-BMSC sheet constructs. Upon implantation of these constructs into 8-mm-diameter critical-sized calvarial bone defects in osteoporotic rats, the BMSC sheets survived in the scaffold and participated in new bone formation.^89^ Devitalised allografts wrapped in mouse BMSC sheets were transplanted into 4-mm critical-sized murine femoral segmental bone defects by Long et al., who showed that the BMSC sheets improved bone callus formation during allograft healing in bone defects and enhanced allograft osseointegration.^90^

**Fig. 3:**
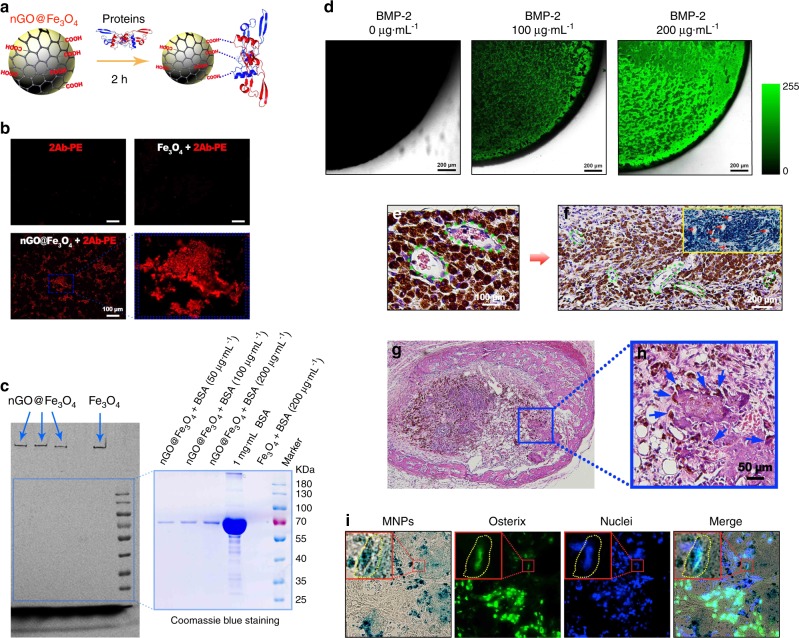
The construction and application of osteogenic microtissues comprising growth-factor-immobilised cell sheets via magnetic force. **a** Proteins and nGO@Fe_3_O_4_ magnetic nanoparticles (MNPs) were incubated to allow binding. **b** The combination of nGO@Fe_3_O_4_ MNPs and 2Ab-PE was detected. **c** The binding ability of nGO@Fe_3_O_4_ MNPs and bovine serum albumin at different concentrations was assessed. **d** The binding of nGO@Fe_3_O_4_ MNPs and bone morphogenetic protein 2 (BMP-2) at different concentrations was detected. **e** The blood vessels were observed to grow into the dental pulp stem cell (DPSC) sheet after subcutaneous implantation in nude mice for 1 week. **f** After implantation for 1 month, blood vessels were observed, and the larger vessels were labelled. **g** After implantation for 1 month, new bone formation was observed around the BMP-2-immobilised cell sheets. **h** The labelled cells were observed to migrate to and participate in the newly formed bone. **i** The nGO@Fe_3_O_4_ MNP-labelled DPSCs were directly observed to differentiate into osteoblasts. (Adapted from ref. ^68^ with permission.)

Growth factors play a key role in tissue regeneration due to their excellent regenerative potential in various tissues, such as bone and cartilage.^91^ Recently, growth factors have been utilised with cell sheets to promote bone regeneration. The platelet-rich plasma (PRP) gel contains several growth factors, including platelet-derived growth factor (PDGF), transforming growth factor (TGF-β1, TGF-β2), insulin-like growth factor (IGF-1, IGF-2) and vascular endothelial growth factor. Qi et al. combined rat BMSC sheets prepared by the mechanical system with the PRP gel and CaP particles and then transplanted the constructs into cortical bone defects in rat femurs. They discovered that incorporating PRP gel/CaP particles into BMSC sheets enhanced bone regeneration.^92^ Unlike the intramembranous ossification-based bone tissue engineering strategy, which mimics the embryological process and requires the immediate establishment of initial vascular networks for implant survival, the endochondral ossification-based bone tissue engineering strategy utilises engineered cartilage as a transient template that can survive in an avascular environment and is capable of inducing angiogenesis via hypertrophic chondrocytes to promote bone regeneration and fracture healing.^93^ Bioactive microparticles capable of not only the controlled delivery of TGF-β1 early to induce cartilage formation but also the sustained delivery of bone morphogenetic protein 2 (BMP-2) to promote bone remodelling were engineered by Dang et al. Human BMSC sheets combined with such microparticles containing growth factors were implanted into rat critical-sized calvarial bone defects and were shown to accelerate bone defect healing.^94^ Our group discovered that nGO@Fe_3_O_4_ MNPs are capable of binding and delivering proteins via the plentiful carboxyl groups provided by the nGO coating (Fig. 3a). The ability of nGO@Fe_3_O_4_ MNPs to bind proteins was verified by incubation with phycoerythrin-labelled secondary antibody (2Ab-PE) and different concentrations of bovine serum albumin (Fig. 3b, c). We were able to utilise nGO@Fe_3_O_4_ MNPs to bind BMP-2 in a concentration-dependent manner (Fig. 3d). Novel scaffold-free osteogenic microtissues comprising cell sheets with immobilised growth factor were constructed by attracting nGO@Fe_3_O_4_ MNP-labelled DPSCs and nGO@Fe_3_O_4_ MNP-bound BMP-2 with magnetic force. Upon subcutaneous implantation of BMP-2-loaded DPSC sheets into nude mice, host blood vessels grew into the microtissue, new bone formed around the microtissue and the nGO@Fe_3_O_4_ MNP-labelled DPSCs differentiated into osteoblasts (Fig. 3e, f).^68^

Vascularisation is essential for the repair of large bone defects. Recently, strategies consisting of co-culturing cell sheets with vascular endothelial cells, stacking vascular endothelial cell sheets with other types of cell sheets or even inserting vascular bundles in cell sheet scaffold constructs have been utilised to construct cell sheets capable of promoting vascularised bone regeneration. Mechanical or temperature-responsive systems were used to prepare the cell sheets. Asakawa et al. created three-dimensional (3D) stratified tissues by stacking cell sheets with HUVEC sheets and discovered that pre-vascular networks composed of HUVECs formed tubular structures, similar to those of native microvasculature in vitro.^95^ Ren et al. created a highly pre-vascularised 3D cell sheet construct by seeding HUVECs on human BMSC sheets and then folding the sheets into a 3D structure. Subcutaneous implantation of the constructs into immunodeficient mice revealed that the pre-vascularised constructs promoted blood vessel formation and functional anastomosis with the host vasculature.^96^ This research group further created a composite construct consisting of an inner highly pre-vascularised cell sheet, which was formed by seeding HUVECs on undifferentiated human BMSC sheets, and an outer osteogenic layer, which was formed by human BMSCs after osteogenic induction. The undifferentiated human BMSC sheets improved the alignment of HUVECs in vitro and promoted the formation of vascular-like networks; in addition, the seeded HUVECs rearranged the ECM secreted by human BMSCs. Subcutaneous implantation of the composite constructs into nude mice resulted in rapid vascularisation and anastomosis with the host vasculature, as well as the formation of functional blood vessels.^97^ Pericytes located within the basement membrane of capillaries and postcapillary venules have a great impact on blood vessel wall stabilisation, endothelial cell proliferation and migration and microvascular blood flow regulation.^98^ A scaffold-free construct was created by combining human BMSC sheets after osteogenic induction with HUVECs and perivascular-like (CD146+) cells, which were induced from human BMSCs. Subcutaneous implantation of the construct into nude mice revealed its excellent osteogenic and angiogenic potential.^99^ A double cell sheet complex was formed by combining an osteogenic cell sheet with a vascular endothelial cell sheet, with both sets of cells induced from rabbit adipose-derived MSCs. Subcutaneous implantation of this complex into nude mice showed that it had an excellent ability to promote osteogenesis and angiogenesis. In addition, the combination of the double cell sheet complex with a coral hydroxyapatite (HA) scaffold resulted in the synergistic promotion of both osteogenesis and blood vessel formation.^100^ A novel vascularised tissue-engineered bone scaffold was developed by wrapping osteogenic matrix cell sheets induced from rat BMSCs around a microporous β-TCP scaffold with a side groove. Subcutaneous implantation of the sheet scaffold constructs into rats with femoral vascular bundles passing through the groove promoted vascularisation and new bone formation.^101^ Another study used an arteriovenous loop (AVL) to circle a β-TCP scaffold by insertion into the lateral groove. Then the construct was wrapped in a rabbit BMSC sheet and transplanted into the thigh muscle pockets of rabbits. New bone formation and vascularisation were promoted by the combination of a BMSC sheet, β-TCP scaffold and AVL.^102^

**Fig. 4:**
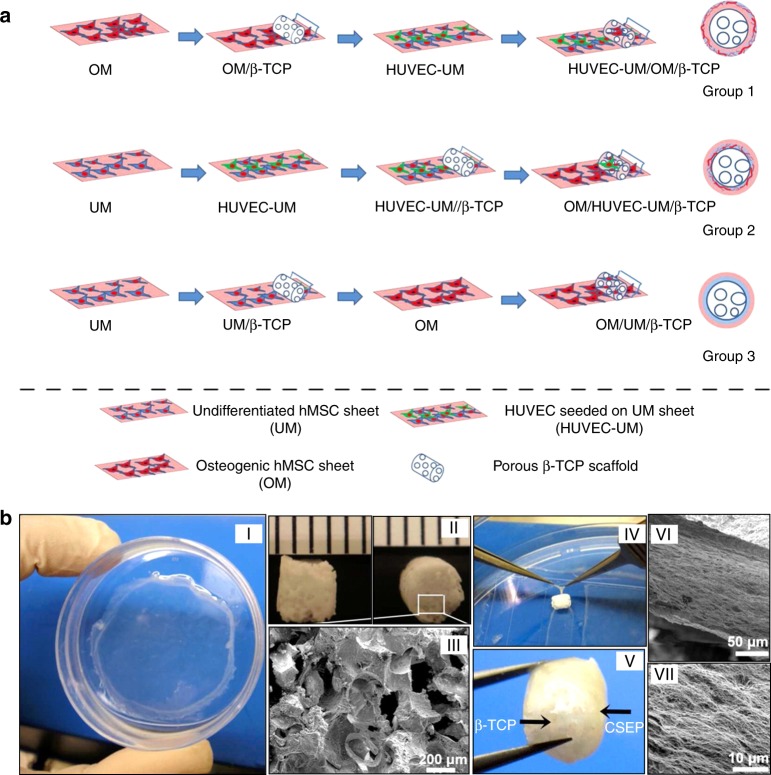
Procedures for preparing cell sheet/beta tricalcium phosphate (β-TCP) composite grafts. **a** Preparing three cell sheet/β-TCP grafts including OM/UM/β-TCP, OM/HUVEC-UM/β-TCP, and HUVEC-UM/OM/β-TCP. **b** Macroscopic view of an human mesenchymal stem cell sheet on a dish (I) and a porous β-TCP scaffold (II). The scanning electron microscopic (SEM) image demonstrates the morphology of β-TCP pores (III). Point forceps were used to wrap the cell sheet onto a β-TCP scaffold (IV), thus generating a HUVEC-UM/OM/β-TCP graft (V). The SEM images show a very dense extracellular matrix of cell sheets on a β-TCP scaffold (VI, VII). (Adapted from ref. ^62^ with permission. Further permissions related to the material excerpted should be directed to the ACS: https://pubs.acs.org.ccindex.cn/doi/10.1021/am502056q.)

The periosteum is a thin membrane that covers the outer surface of bones and plays a significant role in bone development and regeneration. The periosteum is mainly composed of an outer fibrous layer that contains fibroblasts, collagen fibres and blood vessels and nourishes the inner bone and an inner osteogenic layer that contains MSCs and osteoprogenitor cells and is responsible for bone development and regeneration.^103^ Multilayered cell sheets have been prepared using mechanical systems to promote vascularised bone regeneration by mimicking the native periosteum structure. Kang et al. seeded HUVECs on undifferentiated human MSC sheets to mimic the fibrous layer of native periosteum, while an osteogenic human MSC sheet was cultured to mimic the osteogenic layer of native periosteum. Combining these two cell sheets and wrapping them around a β-TCP scaffold resulted in a biomimetic, cell sheet-based engineered periosteum. Subcutaneous implantation of the cell sheet scaffold construct into nude mice enhanced angiogenesis, functional anastomosis with the host vasculature and osteogenesis (Fig. 4).^62^ Zhang et al. combined undifferentiated rat BMSCs with induced endothelial-like cells differentiated from rat BMSCs to form a pre-vascularised cell sheet mimicking the fibrous layer of native periosteum. An osteogenic cell sheet differentiated from rat BMSCs mimicking the osteogenic layer of native periosteum was wrapped within the pre-vascularised cell sheet to form a biomimetic periosteum. Then a porous β-TCP scaffold was wrapped within the biomimetic periosteum and transplanted into rat calvarial defects, wherein the scaffold promoted the formation of blood vessels and new bone tissue.^104^

### Progress in the application of cell sheet technology in articular cartilage and bone–cartilage complex regeneration

Articular cartilage is a highly organised, resilient connective tissue that covers the surface of bone.^105^ Owing to the lack of a blood supply and the low density of chondrocytes, self-repair of articular cartilage is difficult once the latter is damaged.^2^ In recent years, cell sheet technology has shown great potential for cartilage regeneration.^106^ Compared with monolayers, multilayered chondrocyte sheets showed high expression levels of chondrogenic-related genes and proteins, as well as of cell adhesion-related genes and proteins, indicating that multilayered chondrocyte sheets closely mimic native cartilage and might have great ability to regenerate cartilage.^107^ Moreover, multilayered chondrocyte sheets were able to secrete high levels of humoural factors, such as TGF-β and prostaglandin E2, which play key roles in cartilage regeneration.^108^ Multilayered rabbit chondrocyte sheets were prepared by the temperature-responsive system and transplanted into partial-thickness cartilage defects in rabbit femoral condyles at a depth of <1 mm. The multilayered chondrocyte sheets were capable of maintaining the cartilage phenotype and both attaching to and covering the cartilage defects to protect proteoglycans from catabolic factors in the joint.^109^ Yano et al. identified a novel small thienoindazole derivative, TD-198946, by screening 2500 natural and synthetic small compounds for cartilage regeneration. TD-198946 was able to induce chondrogenic differentiation and abundant ECM production while simultaneously suppressing hypertrophic differentiation and endochondral ossification; therefore, regenerated cartilage could be maintained without being replaced by bone.^110^ By using the temperature-responsive system, multilayered chondrocyte sheets were created using mouse chondrocytes or canine costal dedifferentiated chondrocytes and human BMSC sheets treated with TD-198946 and then transplanted into defects in the femoral condylar cartilage of mice (1 mm in diameter), canines (3 mm in diameter and 1 mm in depth) and NOD/SCID mice (1 mm in diameter). The transplanted cell sheets promoted cartilage regeneration without causing chondrocyte hypertrophy.^43^

In addition to the destruction of articular cartilage, the destruction of subchondral bone is usually involved in osteoarthritis.^111^ Therefore, the repair of bone–cartilage complex defects has received much attention in the field of regenerative medicine, and many researchers have applied the cell sheet technology for the treatment of bone–cartilage complex defects. Multilayered chondrocyte sheets prepared by the temperature-responsive system were autologously transplanted into full-thickness femoral condylar cartilage defects 6 mm in diameter and 5 mm in depth in minipigs by Ebihara et al., who discovered that the multilayered chondrocyte sheets promoted cartilage repair and regeneration. However, in some cases, the effect on subchondral bone regeneration was unsatisfactory.^20^ Multilayered chondrocyte sheets combined with synovial cells using the temperature-responsive system were transplanted into osteochondral defects 5 mm in diameter and 3 mm in depth in the femoral patellar groove of rabbits by Ito et al., who reported increased repair and regeneration of both cartilage and subchondral bone in bone–cartilage complex defects.^44^ Wang et al. utilised minipig-derived chondrocyte sheets prepared by the mechanical system to cover PCL/HA scaffolds seeded with minipig-derived BMSCs. Subcutaneous implantation of the constructs into nude mice regenerated condyle-shaped osteochondral composites.^64^ In addition to immunodeficient animal models, minipigs have been used to show that subcutaneous and intramuscular construct transplantation results in the regeneration of biological condyles with an osteochondral construction similar to that of native condyles.^22^ Takaku et al. used the temperature-responsive system to prepare multilayered cell sheets consisting of both chondrocytes and synovial cells from firefly luciferase-expressing transgenic rats and transplanted them into wild-type rats. The transplanted cell sheets could remain and survive in osteochondral defects in the knee joint for up to 21 months, which confirmed the effects of the cell sheets on the repair and regeneration of bone–cartilage complex defects.^112^ The mechanism of cell sheet-mediated repair of bone–cartilage complex defects was discovered by Shimizu et al. via cell tracking experiments using green fluorescent protein (GFP) transgenic rats. Moreover, these researchers found that higher expression of TGF-β1 in chondrocyte sheets might contribute to cartilage repair.^113^

**Fig. 5:**
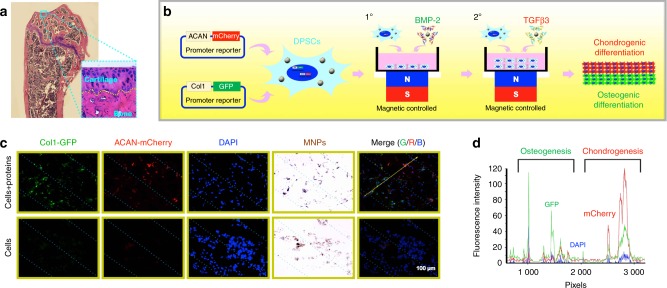
The construction and application of osteochondral microtissues composed of chondrogenic cell sheets and osteogenic cell sheets based on the magnetically controlled approach. **a** The structure of the normal mouse knee joint with the cartilage–bone interface labelled by a yellow dotted line. **b** Schematic illustration of the process used to construct an integrated osteochondral tissue via the magnetically controlled method. **c** After implantation for 1 week, mCherry+ chondrocytes and GFP+ osteoblasts were observed using a fluorescence microscope. **d** The fluorescence intensity along the yellow arrow was recorded. (Adapted from ref. ^68^ with permission.)

Our group constructed microtissues consisting of chondrogenic cell sheets and osteogenic cell sheets for the repair of bone–cartilage complex defects based on the magnetically controlled approach. The osteogenic growth factor BMP-2 and the chondrogenic growth factor TGF-β3 were bound to nGO@Fe_3_O_4_ MNPs. Magnetically attracting nGO@Fe_3_O_4_ MNP-labelled DPSCs and nGO@Fe_3_O_4_ MNP-bound growth factors led to the creation of microtissues with a closely integrated double-layered structure mimicking the cartilage–bone interface (Fig. 5a), in which the upper layer was DPSC sheets with immobilised TGF-β3 and the lower layer was DPSC sheets with immobilised BMP-2 (Fig. 5b). One week after the subcutaneous implantation of these microtissues into nude mice, DPSCs in the upper and lower layers were observed to have differentiated into mCherry+ chondrocytes and GFP+ osteoblasts, respectively (Fig. 5c, d).^68^

## Challenges in applying the cell sheet technology in bone and cartilage regeneration and potential research areas

In recent decades, much progress has been made in the preparation and application of cell sheets. With the efforts of many researchers, cell sheet technology is becoming a promising strategy for the repair and regeneration of bone and cartilage defects. However, some obstacles remain in the application of cell sheet technology. For example, constructing tissue or organ-like tissue with a complicated structure and morphology is difficult, and the constructed cell sheets usually lack mechanical properties. The cells in multilayered cell sheets are prone to necrosis. In addition, the components of cells and ECM in cell sheets are quite different from those in native cartilage.

To construct 3D bone and cartilage, the cells or cell sheets must be stacked to form complex structures and shapes. Various systems are available for constructing cell sheets. Temperature-responsive and mechanical approaches are the most widely used to prepare cell sheets for bone and cartilage regeneration, likely because temperature-responsive systems are the most traditional and widely studied systems and because mechanical approaches are easy due to the lack of a need for specific culture substrates or techniques. The stacking technique is widely utilised for the preparation of multilayered cell sheets. With the application of magnetic technology, multilayered cell sheets can be prepared by magnetically attracting MNP-labelled cells layer by layer in a single step. Cell patterning and micropatterning technology has been introduced to modify the morphology of cell sheets. In a magnetic system, the shape of cell sheets can be precisely controlled using different numbers of magnets or magnet patterns. However, using existing cell sheet preparation techniques to prepare relatively more complex tissues and even organs is still challenging. Thus existing preparation systems need to be further improved, or novel effective preparation systems need to be proposed.

The maintenance of a sufficient nutrient and oxygen supply and the efficient exchange of cellular metabolites contribute to the survival of cells in cell sheets. Cells deep in thick multilayered cell sheets are prone to necrosis during application for bone tissue regeneration due to the insufficient nutrient and oxygen supply and the poor exchange of cell waste. Vascularisation is of great importance for bone tissue regeneration. The rapid growth of blood vessels into transplanted cell sheets can supply nutrients and oxygen and exchange metabolites in a timely manner, thus effectively promoting the survival of transplanted cells and the growth of host tissues. The rapid vascularisation of cell sheets is an important research direction for bone regeneration.

Native cartilage is composed of a large amount of ECM components and a small number of chondrocytes. However, currently prepared cell sheets usually contain a large number of cells with a relatively small proportion of ECM and are quite different from native cartilage. Recently, a micropatterned collagen sheet was fabricated by the permeation of a piece of doughnut-shaped paper by a cell–collagen mixture, and the components of this sheet are more similar to native cartilage than the other cell sheets mentioned above.^114^ Constructing cell sheets with a composition similar to that of native cartilage is crucial; however, further research is needed for the application of such sheets in cartilage regeneration.

## Supplementary information

A permission of Advanced Materials

A permission of Advanced Functional Materials

A permission of ACS Applied Materials & Interfaces
