# Supplementary material for: Recent advances in cell sheet technology for bone and cartilage regeneration: from preparation to application
Source: Int J Oral Sci. 2019 May 21;11(2):17. doi: 10.1038/s41368-019-0050-5 (PMC6527566; doi:10.1038/s41368-019-0050-5)
Supplement: Supplementary file 3 — A permission of ACS Applied Materials & Interfaces [file 41368_2019_50_MOESM3_ESM.pdf]

Dear Dr. Jiang:

Thank you for contacting ACS Publications Support.

Your permission request is granted and there is no fee for this reuse. In your planned reuse, you must cite the ACS article as the source, add this direct link (https://pubs.acs.org.ccindex.cn/doi/10.1021/am502056q) and include a notice to readers that further permissions related to the material excerpted should be directed to the ACS.

I hope this information helped. Please let me know if I can be of further assistance.

Sincerely,

Kryxie J. Ramirez
ACS Customer Services & Information
https://help.acs.org/
